# Excessive Worrying as a Central Feature of Anxiety during the First COVID-19 Lockdown-Phase in Belgium: Insights from a Network Approach

**DOI:** 10.5334/pb.1069

**Published:** 2021-12-30

**Authors:** Alexandre Heeren, Bernard Hanseeuw, Louise-Amélie Cougnon, Grégoire Lits

**Affiliations:** 1 Psychological Sciences Research Institute, Université catholique de Louvain, Louvain-la-Neuve, Belgium; 2 Institute of Neuroscience, Université catholique de Louvain, Brussels, Belgium; 3 Neurology Department, Cliniques Universitaires Saint-Luc, Brussels, Belgium; 4 Gordon Center for Medical Imaging, Radiology Department, Massachusetts General Hospital, Harvard Medical School, Boston, MA, USA; 5 Media Innovation & Intelligibility Lab, Université catholique de Louvain, Louvain-la-Neuve, Belgium; 6 Language and Communication Institute, Université catholique de Louvain, Louvain-la-Neuve, Belgium

**Keywords:** COVID-19, Anxiety, Worry, GAD, Network Approach to Psychopathology, Directed acyclic graph, Gaussian Graphical Model, Psychopathology, Pandemic, Lockdown

## Abstract

Since the WHO declared the COVID-19 pandemic on March 11, 2020, the novel coronavirus, SARS-CoV-2, has profoundly impacted public health and the economy worldwide. But there are not the only ones to be hit. The COVID-19 pandemic has also substantially altered mental health, with anxiety symptoms being one of the most frequently reported problems. Especially, the number of people reporting anxiety symptoms increased significantly during the first lockdown-phase compared to similar data collected before the pandemic. Yet, most of these studies relied on a unitary approach to anxiety, wherein its different constitutive features (i.e., symptoms) were tallied into one sum-score, thus ignoring any possibility of interactions between them. Therefore, in this study, we seek to map the associations between the core features of anxiety during the first weeks of the first Belgian COVID-19 lockdown-phase (*n* = 2,829). To do so, we implemented, in a preregistered fashion, two distinct computational network approaches: a Gaussian graphical model and a Bayesian network modelling approach to estimate a directed acyclic graph. Despite their varying assumptions, constraints, and computational methods to determine nodes (i.e., the variables) and edges (i.e., the relations between them), both approaches pointed to excessive worrying as a node playing an especially influential role in the network system of the anxiety features. Altogether, our findings offer novel data-driven clues for the ongoing field’s larger quest to examine, and eventually alleviate, the mental health consequences of the COVID-19 pandemic.

Epidemic-related lockdowns are known to yield severe and long-lasting consequences on mental health (for a systematic review, see Brooks et al., 2020), and the COVID-19 pandemic is no exception to this statement. Early data indicated that the COVID-19 pandemic had indeed impacted mental health (for reviews, see Asmunsdon & Taylor, 2020; Cénat et al., 2021; Xiong et al., 2020; Wu et al., 2021), with anxiety symptoms being one of the most frequently reported problems (e.g., Mertens et al., 2020; Qiu et al., 2020; Xiong et al., 2020).

In Belgium, the number of people reporting anxiety symptoms increased substantially during the first lockdown of March 2020 compared to similar data collected two years before (Sciensano, 2020). Similar findings have been reported across different countries (e.g., González-Sanguino et al., 2020; Zhao et al., 2020). And that should not come as a surprise. The profound health, social, and economic consequences of the COVID-19 lockdown and other social distancing measures are likely to be anxiogenic for many, regardless of whether they have had direct exposure to the virus or not (Heeren, 2020; Park, Velez, Kannan, Chorpita, 2020). For instance, the threat of infection posed by the virus may trigger worry about significant others’ infection and death (e.g., Mertens et al., 2020; Schimmenti, Billieux, & Starcevic, 2020). Likewise, the potential economic consequences of the lockdown phases and social distancing measures may foster one’s worry about the coming economic recession, reduced purchasing power, and risk of jobs loss for oneself and one ‘significant others (e.g., Blix et al., 2020; Cook & Grimshaw, 2021; Fana, Torrejón Pérez, & Fernández-Macías, 2020; Tamesberger & Bacher, 2020).

The above illustrations exemplify the notion of excessive worry (apprehensive expectation), one of the cardinal features of General Anxiety Disorder (GAD). The DMS-5 (American Psychiatric Association [APA], 2013) outlines that the distinction between excessive and non-pathological worry is that excessive worries are difficult to stop or control and are perceived as distressing and pervasive in terms of occurrence, duration, and variety of worry topics (Garcia, Renna, & Mennin, 2015; Newman et al., 2013). Moreover, excessive worry has been reported to be associated with discomforting and disruptive anxiety and physical symptoms, like restlessness, irritability, trouble relaxing, and muscle tension, which also are distressing and impairing (for reviews, see Garcia, Renna, & Mennin, 2015; Newman et al., 2013).

With respect to the COVID-19 pandemic, representative cohort studies, comparing data collected before the pandemic with those of the first weeks of the first COVID-19 lockdown-phase, reported a worldwide rise in the proportion of people experiencing clinically significant levels of GAD symptoms (Gao et al., 2020; Islam, Ferdousa, & Potenza, 2020; for a systematic review, see Salari et al., 2020). However, most of these studies relied on a unitary approach to GAD by tallying its different constitutive features (i.e., symptoms) into one sum-score, thus obscuring the role each specific symptom may play individually in regard to the other ones. And this is unfortunate as there have been remarkable strides toward analyzing mental disorders’ symptoms individually to understand their function better. For instance, in GAD research, excessive worry could trigger trouble relaxing, and vice versa (e.g., Newman et al., 2019).

Recently, a network approach to psychopathology has appeared. In this approach, mental disorders are conceptualized as network systems of interacting symptoms. From this perspective, instead of investigating mental disorders as reflecting a single, unitary construct, one can examine the structure of, and associations between, the symptoms themselves. Investigating a mental disorder’s network structure can thus grant new insight into its symptoms-to-symptoms associations and topology (McNally, 2021). According to this perspective, symptoms, therefore, possess independent causal powers that can influence other symptoms (e.g., excessive worrying can trigger muscle tension; difficulty to control worry can motivate indecisiveness); they are not merely passive indicators of an underlying disease. Hence, in this conceptual framework, symptoms are constitutive, not reflective, of disorder (Borsboom and Cramer, 2013; McNally, 2021).

Though only recently pioneered by Borsboom and his colleagues (e.g., Borsboom, 2017; Borsboom et al., 2011, Borsboom & Cramer, 2013), this approach has quickly become a hot topic in contemporary clinical psychology. Many studies have thus accordingly used this framework to investigate the interrelations between systems of symptoms and speculate as to the clinical implications (for a general overview, see Blanchard & Heeren, 2022; McNally, 2021; for systematic reviews, see Contreras et al., 2019; Robinaugh et al., 2019).

Moreover, the use of network theory to model psychopathology allows the adoption of network tools and graph theory concepts to understand mental disorders. One of these network concepts is node centrality. The core tenet of this latter is that some nodes (here, symptoms) in the network are more important to the network structure than others (e.g., Blanchard & Heeren, 2022; McNally, 2021). Nodes are viewed as more central if they are especially connected to the rest of the network (i.e., sharing many strong connections with other nodes), because they can then influence the entire network. In network models, highly central nodes are thought to maintain the network structure since, once activated, they can quickly spread that activation to the entire system (e.g., Barabási and Albert, 1999; Borgatti, 2005). Within the network approach to psychopathology, central nodes have accordingly been theoretically (Borsboom & Cramer, 2013) and empirically (e.g., Elliott, Jones, & Schmidt, 2020; Papini, Rubin, Telch, Smits, & Hien, 2020) viewed with respect to the prognosis of a disorder. Regarding GAD, previous network research has identified excessive worry and uncontrollability of worry as the most central nodes in the network structure of GAD symptoms among clinical (e.g., Beard et al., 2016; Hsu et al., 2020) and subclinical (e.g., Osborn et al., 2020) samples.

Concerning the COVID-19 pandemic, only a few studies have so far applied a network analytic framework to examine the interdependence between different factors assumedly involved in the psychological responses to the pandemic, and especially regarding the fear of infection or the anxious response to the structural impact of the pandemic (e.g., Jiang et al., 2020; Papini et al., 2020; Taylor et al., 2020). And for the anxiety symptoms reported in the general population during the COVID-19 pandemic, only two studies (i.e., Hoffart et al., 2021; Wang et al., 2020) have, until now, investigated the interrelationships between the distinct hallmark symptoms of GAD as experienced during lockdown through the lens of the network approach. For ease of comparison, both studies (i.e., Hoffart et al., 2021; Wang et al., 2020) relied on the Generalized Anxiety Disorder-7 (GAD-7; Spitzer et al., 2006), one of the most commonly used self-reported screening tools in epidemiological and clinical research for assessing GAD symptoms (e.g., Schalet et al., 2014). In their study, Wang et al. (2020) identified excessive worry, trouble relaxing, and restlessness as the most central nodes in the structure of GAD symptoms among Chinese adults during the early first weeks of the outbreak (i.e., lockdown). Likewise, Hoffart et al. (2021) identified excessive worry, uncontrollability of worry, and trouble relaxing (but not restlessness) as the most central nodes among Norwegian adults during the early weeks of the lockdown.

In this project, we thus seek to map GAD symptoms’ network structure at the early stages of the first weeks of the first Belgian COVID-19 lockdown-phase. To do so, we implemented the network computational tools via a preregistered reanalysis of an existing dataset (i.e., Lits et al., 2020) that includes the assessment of GAD symptoms via the GAD-7—as in Hoffart et al. (2021) Wang et al. (2020)— in a large and representative sample of participants living in the French-speaking part of Belgium who took part to an online survey conducted during the first weeks following the first Belgian lockdown (i.e., late March 2020).

Especially, we had three main goals (see also our formal preregistration; *https://osf.io/p3v4e*). First, following Hoffart et al. (2021) and Wang et al. (2020), we endeavored to clarify the pairwise connections among the distinct GAD symptoms. To this end, we computed a graphical Gaussian model (GGM) model, an undirected network model wherein the edges represent conditional independent relationships between nodes (Epskamp & Fried, 2018). Relatedly, we also quantified each node’s importance to the resulting network structure via the computation of centrality metrics (Costantini et al., 2015; Opsahl, Agneesens, Skvoretz, 2010). Second, we tested whether the distinct constitutive features/symptoms of GAD emerge as a single network system, or whether they instead organize into distinct subnetworks or communities of nodes (i.e., cluster of symptoms). And, if so, whether certain nodes function as *bridges* linking these communities (Jones et al., 2019).

Finally, we followed recent publications (e.g., Bernstein et al., 2017; Blanchard et al., 2021a; Heeren et al., 2020; Kalkan et al., 2021; McNally et al., 2017) and relied on Bayesian network methods to estimate a directed acyclic graph (DAG), which encodes the conditional independence relationships between the variables of interest and characterizes their joint probability distribution (McNally, 2016; Pearl et al., 2016; Scutari, 2010). Hence, the resulting network is directed and possesses arrows reflecting the predicted direction of the probabilistic dependence between nodes (McNally, 2016; Moffa et al., 2017). Although DAGs were not estimated in previous research on the impact of COVID-19 lockdown on anxiety, we decided to rely on DAGs to further examine the probabilistic dependencies between the distinct symptoms and generate a data-driven probabilistic computational model of the organization of the GAD symptoms during the COVID-19 lockdown.

## Method

### Preregistration and Open Science Practices

As this is an exploratory study using secondary data, we followed the recent guidelines for increasing the transparency of analysis of preexisting data set (Weston et al., 2019) and preregistered the analysis plan^1^ and prior knowledge regarding the dataset at *https://osf.io/p3v4e*. Our R code and de-identified data are available at *https://osf.io/9ehja/*.

### Participants

We reanalyzed data from a previous study (i.e., Lits et al., 2020). Participants were recruited from the general community via online social media as well as online, radiophonic, and paper news coverage. The sample depicted in the initial study was built upon a quota sampling method to align with the characteristics (i.e., age, education level, and gender) of the population living in the French-speaking part of Belgium over the age of 16 (following the results of the STATBEL 2019 governmental survey on Belgian workforce). Further details about the data collection can be found in the initial study (i.e., Lits et al., 2020). This recruitment procedure resulted in a sample of 2,829 Belgian French-speaking participants (69% women, 30,4% men, 0.2% others; and .4 preferring not disclosing their gender) between 16 and 91 years old (*Median* = 42; *M* = 44.50, *SD* = 17.19). The initial study was approved by the Ethical Committee of the Louvain School of Political and Social Sciences (UCLouvain, Belgium) and conducted according to the Declaration of Helsinki. Each participant provided informed consent before completing the survey.

### Measures

The Generalized Anxiety Disorder-7 (GAD-7; Spitzer et al., 2006) is a widely used, 7-item scale wherein each item covers of the DSM-IV criteria for GAD. Participants rate each item on a 4-point Likert-type scale, from 0 (*Never*) to 3 (*Almost every Day*). For each item, higher score denotes greater endorsement of the symptoms targeted by the item. We used the validated French self-report version of the scale (Micoulaud-Franchi et al., 2016). The internal reliability of GAD-7 was high in the present sample, with a Cronbach’s alpha of .89 for the global scale score. The mean total GAD-7 score in the present sample was 4.92 (*SD* = 4.68, *Min* = 0, *Max* = 21), with 23.05% of the participants exhibiting a score equal to or higher than the cut-off score of 8 recommended to identify individuals likely (i.e., with a sensitivity of 92% and specificity of 76%) to qualify for the diagnosis of generalized anxiety disorder (Kroenke et al., 2007; Plummer et al., 2016). Among our total sample, 4.45% had a total score denoting severe symptomatology (i.e., total scale score strictly higher than 15; and 29.76% had score reflecting mild-to-moderate (i.e., score ranging between 5 and 14) symptomatology (Kroenke et al., 2007; Plummer et al., 2016).

### Data Analysis Strategy

#### Data Preparation

Although none of the variables violated normality according to benchmarks of skewness between –2 to + 2 and kurtosis between –7 to +7 (Curran et al., 1996), we followed guidelines in psychological network analyses (Epskamp & Fried, 2018) and applied the nonparanormal transformation to our seven variables of interest via the R package *huge* (Jiang et al., 2019).^2^

#### Check for potential nodes redundancy

Because some of our variables may overlap conceptually (e.g., the items denoting “*Worrying too much about different things* » and « *Feeling afraid as if something awful might happen* »), we implemented a data-driven method to confirm that none of our seven variables (i.e., the seven GAD-7’s items) were redundant. To do so, we followed the procedure described in recent publications (e.g., Bernstein et al., 2019; Everaert & Joormann, 2019; Heeren & McNally, 2018). We first tested whether our correlation matrix was positive definite. Had a non-positive definite matrix emerged, this would reflect that our variables were a mere linear combination of other variables. We then implemented the Hittner method (Hittner et al., 2003) to search for potential highly inter-correlated (r > 0.50) pairs of variables that also correlated to the same degree with other variables (i.e., > 75% of correlations with other variables did not significantly differ for a given pair). The Hittner method was implemented via the goldbricker function of the R package *networktools* (Jones, 2018). This method did not identify any redundancy between our seven variables.

#### Graphical LASSO Network

We present a GGM that was regularized through the graphical LASSO (Least Absolute Shrinkage and Selection Operator) algorithm, which has two main goals (Friedman et al., 2011). First, it estimated regularized partial correlations between pairs of nodes, thereby excluding spurious associations (or edges) resulting from the influence of other nodes in the network. Second, it shrunk trivially small associations to zero, thus eliminating possibly “false positive” edges from the model and returning a sparser network including only the strongest edges. We did so via the R package *qgraph* (Epskamp et al., 2012, 2018), which automatically implements such a regularization along with model selection based on the Extended Bayesian Information Criterion (EBIC; Foygel & Drton, 2011). This procedure computes 100 models with varying degrees of sparsity; a final model is chosen according to the lowest EBIC value, given a specific hyperparameter gamma (γ), which regulates the trade-off between admitting false-positive edges and suppressing true edges. In general, the hyperparameter γ is set between 0 (favoring a model with more edges) and 0.5 (promoting a simpler model with fewer edges). Following recommendations based on stimulation studies (for details, see Epskamp et al., 2018), we set γ to 0.5 to be confident that our edges are true. To assess the stability of our edge weights, we implemented a nonparametric bootstrapping procedure (with 1,000 bootstrapped samples with replacement) to bootstrap the edge weights’ confidence regions. Using a bootstrapped difference test (Epskamp et al., 2018), we also examined whether the edge weights significantly differed from one another.

To gauge each node’s importance in the regularized GGM, we then computed the expected influence centrality indices (Robinaugh et al., 2016). This index quantifies the cumulative importance of each node and describes the sum of the edge weights attached to this node, considering both positive and negative values (Robinaugh et al., 2016). Hence, higher values indicate greater centrality in the network and so higher node’s local connectivity (McNally, 2021). The plot represents the raw expected influence value of each node. Following recent guidelines (Epskamp et al., 2018), we assessed the stability of this metric’s estimates by implementing a person-dropping bootstrap procedure (with 1,000 bootstrapped samples with replacement) and determined the CS-coefficient (details are available in the supplementary materials). Capitalizing on this person-dropping bootstrap procedure, we performed a bootstrapped difference test (Epskamp et al., 2018) to examine whether nodes significantly differ from one another in terms of centrality estimates.

In addition, we also estimated node predictability,^3^ which depicts the proportion of explained variance of a node by all its neighboring nodes in the network (Haslbeck & Fried, 2018). To do so, we relied on the package (Haslbeck & Waldorp, 2017). Node predictability was plotted as a pie chart in the outer ring of each node. Note that predictability across nodes also tells us whether a (part of a) network is primarily determined by itself through strong mutual interactions between nodes (high predictability) or whether it is determined mainly by other factors that are not included in the network (low predictability)—i.e., therefore showing a larger influence from dimensions that are external to the model (for a discussion, see Haslbeck & Waldorp, 2017; McNally, 2021).

Finally, we investigated the GGM’s community structure—that is, whether nodes form a unitary network structure or whether they cluster into distinct sub-networks or communities of nodes—by implementing the Spinglass modularity-based community detection algorithm (Reichardt & Bornholdt, 2006). As in previous studies (e.g., Bernstein et al., 2019; Everaert & Joormann, 2019, Heeren et al., 2018; Robinaugh et al., 2014), we chose this algorithm given its suitability for revealing the community structure of signed networks (Traag & Bruggeman, 2009; Yang et al., 2016), that is networks composed of both positive and negative edge weight values. We implemented this algorithm via the *spinglass.community* function of the R package *igraph* (Csardi & Nepusz, 2006).

#### Directed Acyclic Graph (DAG)

Following previous psychological research (e.g., Bernstein et al., 2017; Blanchard et al., 2021a; Heeren et al., 2020; McNally et al., 2017), we estimated the DAGs via the implementation of a Bayesian hill-climbing algorithm (Scutari, 2010; Scutari & Denis, 2015). To do so, we relied on the R package *bnlearn* (Scutari, 2010; Scutari & Denis, 2015). As implemented in this package, this approach relies on a bootstrap function that estimates the structural features of the model by adding edges, removing them, and reversing their direction to eventually optimize the goodness-of-fit target score, i.e., the Bayesian Information Criterion (BIC; a relative measure of a model’s goodness-of-fit). This bootstrap function requires an iterative procedure of randomly restarting this process with various possible edges connecting various node pairs, disturbing the network system, and applying 50 different random restarts to circumvent local maxima. As in recent implementations of this algorithm (e.g., Bernstein et al., 2017; Blanchard et al., 2021a; Heeren et al., 2020; McNally et al., 2017), we introduced, for each restart, 100 perturbations (i.e., attempts to insert, delete, or reverse an edge). As this iterative process of restart/perturbations unfolds, the algorithm returns the model with the optimal BIC value.

As in recent publications (e.g., Bernstein et al., 2017; Blanchard et al., 2021a; McNally et al., 2017), we then ensured the stability of the resulting DAG as follows. We bootstrapped 10,000 samples (with replacement), estimated a network for each of the bootstrapped 10,000 samples, and ultimately averaged the resulting 10,000 networks to generate a final network structure via a two-step method. First, we determined how frequently a given edge appeared in the 10,000 bootstrapped networks. We then applied the optimal cut-point approach of Scutari and Nagarajan (2013) for retaining edges, which yields networks with both high sensitivity and high specificity. Second, we determined the direction of each surviving edge in the bootstrapped networks. If an edge pointed from node A to node B in at least 51% of the bootstrapped networks, then this direction was reported in the final DAG using an arrow pointing from node A to node B.

For ease of interpretation, we followed prior research (e.g., Bernstein et al., 2017; Blanchard et al., 2021a; Heeren et al., 2020; McNally et al., 2017) and produced two visualizations of the resulting outputs. In the first one, the thickness of the arrow represents the change in the BIC values when that arrow is removed from the network. In this way, the thicker the arrow, the more that arrow contributes to the model structure (McNally et al., 2017). In the second visualization, the thickness of the arrow denotes directional probabilities—that is, the proportion of the bootstrapped networks wherein that arrow was pointing in that direction. In this way, the thicker the arrow, the larger the proportion of bootstrapped networks wherein this edge pointed in the direction depicted.

## Results

Descriptive information (i.e., mean, standard deviation, skewness, kurtosis, and range) are provided in the supplementary materials (see *Table S1)*. The Pearson zero-order correlations between the variables are also provided in the Supplementary Materials (see *Figure S1*).

### The Gaussian graphical model (GGM)

**Figure 1:**
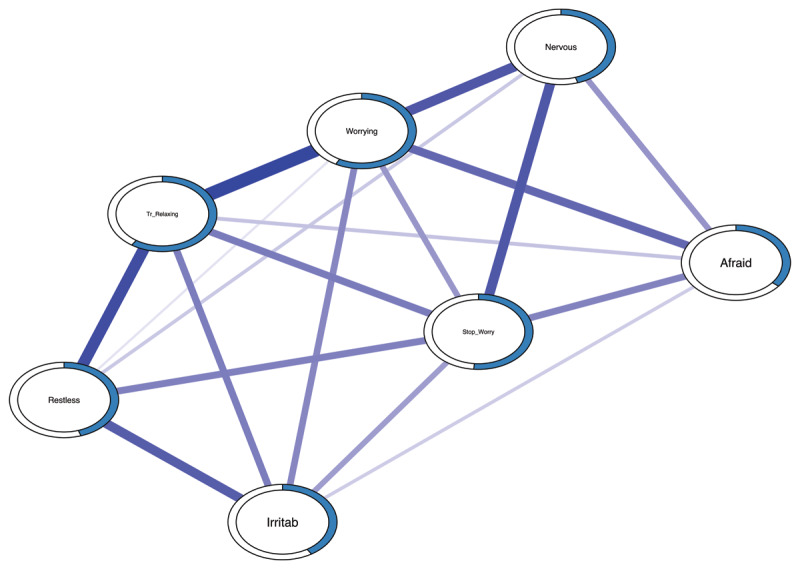
Graphical Gaussian Model Constructed via the Graphical LASSO. ***Note***: The thickness of an edge reflects the magnitude of the association (the thickest edge representing a value of .32). The blue rings around the nodes indicate the proportion of explained variance in that node by all other nodes. Nervous = Feeling nervous, anxious, or on edge (item 1); Stop_Worry = Not being able to stop or control worrying (item 2); Worrying = Worrying too much about different things (item 3); Tr_Relaxing = Trouble relaxing (item 4); Restless = Being so restless that it is hard to sit still (item 5); Irritab = Becoming easily annoyed or irritable (item 6); Afraid = Feeling afraid as if something awful might happen (item 7).

***Figure 1*** depicts the GGM network estimated via the graphical LASSO algorithm.^4^ The thickness of the edge denotes the strength of the association, with a thicker edge denoting a larger association. We used the layout algorithm of Fruchterman and Reingold (1991) to determine node placement, so that nodes closer to the center of the network tend to yield the strongest associations with other nodes. A few associations stood out: “Worrying Too Much” and “Trouble Relaxing” (*r* =.32), “Trouble Relaxing” and “Being Restless” (*r* =.27), “Worrying Too Much” and “Nervousness” (*r* =.23), “Irritability” and “Being Restless” (*r* =.22). We also verified the certainty and precision of the edge weight estimates (see *Figure S4* in the Supplementary Materials). Moreover, the bootstrapped difference test indicated that the strongest edges were significantly larger than most others (see *Figure S5* in the Supplementary Materials).

**Figure 2:**
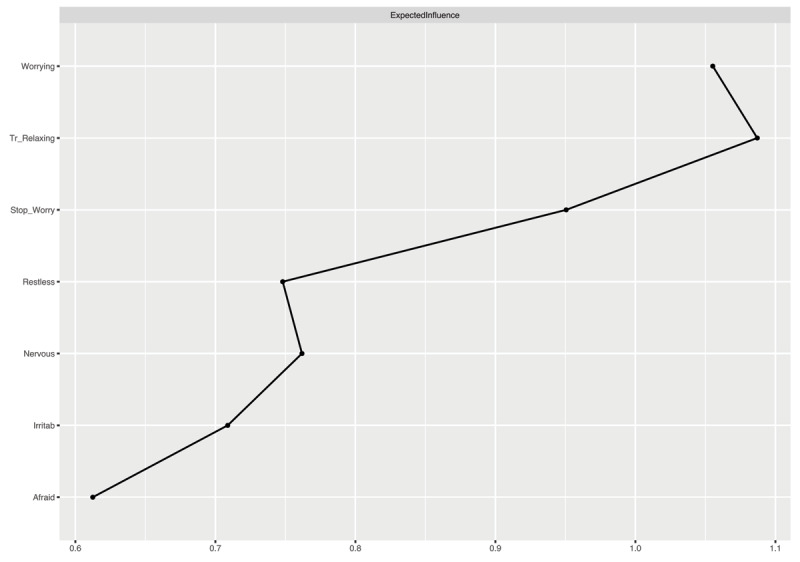
Expected Influence Estimates of the Graphical Gaussian Model Constructed via the Graphical LASSO. ***Note***: Nervous = Feeling nervous, anxious, or on edge (item 1); Stop_Worry = Not being able to stop or control worrying (item 2); Worrying = Worrying too much about different things (item 3); Tr_Relaxing = Trouble relaxing (item 4); Restless = Being so restless that it is hard to sit still (item 5); Irritab = Becoming easily annoyed or irritable (item 6); Afraid = Feeling afraid as if something awful might happen (item 7).

***Figure 2*** depicts the expected influence estimates. Higher values signify greater centrality and, thus, stronger association with other nodes. “Worrying Too Much” and “Trouble Relaxing” were the two nodes yielding the highest expected influence values in the GGM. In contrast, “Feeling Afraid that Something Awful Might Happen” exhibited the lowest expected influence value in the GGM.

The output of the person-dropping bootstrap approach (Epskamp et al., 2018) revealed that the stability of the centrality estimates was high (see *Figure S6* in the Supplementary Materials). Moreover, the bootstrapped different test indicates that “Worrying Too Much” and “Trouble Relaxing” were significantly more central than most other nodes (for more details, see *Figure S7* in the Supplementary Materials). This was also reflected by levels of node predictability (see ***Figure 1***), with most explained variance for Worrying Too Much” (58%) and “Trouble Relaxing” (59%). Note that, on average, most nodes showed relatively high predictability.

Finally, the Spinglass algorithm for community detection did not detect distinct communities (or clusters) of nodes within GGM’s network, indicating that the seven nodes emerged as one single network structure with all nodes belonging to the same community.

### Directed Acyclic Graphs (DAGs)

**Figure 3:**
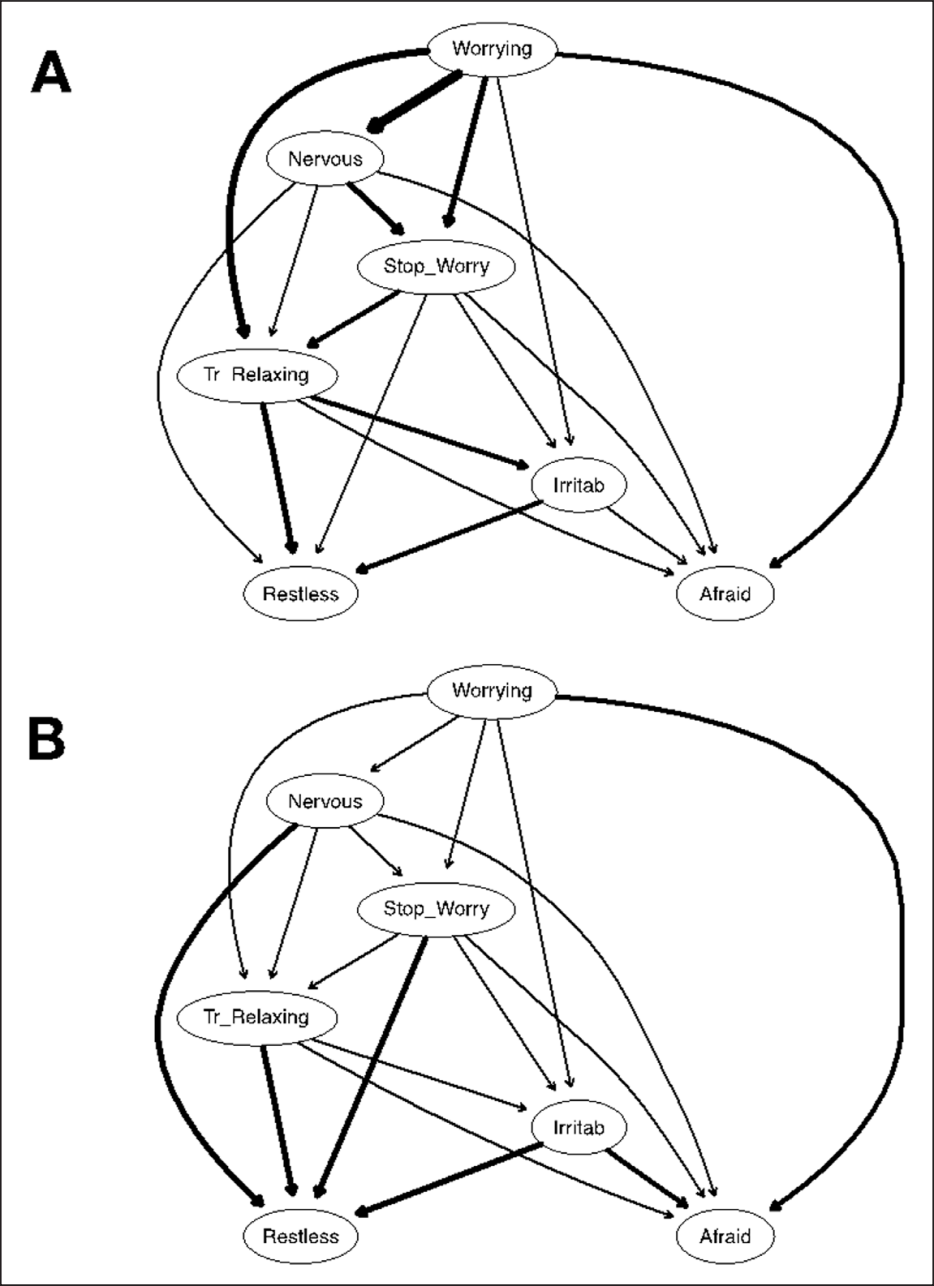
Directed acyclic graphs (DAGs). ***Note***: Panel A: Arrow thickness denotes the importance of that arrow to the overall network model fit. Greater thickness reflects larger contribution to the model fit. Panel B: Arrow thickness indicates directional probability. Greater thickness reflects larger proportions of the bootstrapped networks wherein the arrow pointed in that direction. Nervous = Feeling nervous, anxious, or on edge (item 1); Stop_Worry = Not being able to stop or control worrying (item 2); Worrying = Worrying too much about different things (item 3); Tr_Relaxing = Trouble relaxing (item 4); Restless = Being so restless that it is hard to sit still (item 5); Irritab = Becoming easily annoyed or irritable (item 6); Afraid = Feeling afraid as if something awful might happen (item 7).

***Figure 3*** shows the DAGs resulting from 10,000 bootstrapped samples. In both DAGs, arrows that are present in the graph were retained because their strength was greater than the optimal cut-point resulting from the Scutari and Nagarajan (2013) method.

In ***Figure 3*** (Panel A), arrow thickness denotes the change in the Bayesian Information Criterion (BIC; a relative measure of a model’s goodness-of-fit) when that arrow is removed from the network. In other words, the more an arrow contributes to the model fit, the thicker it is (McNally et al., 2017). The most important arrows to the network structure connect “Worrying Too Much” and “Nervousness” (with a change in BIC of –603.89), “Worrying Too Much” and “Trouble Relaxing” (with a change in BIC of –286.63), and “Worrying Too Much” and “Difficulty to Control Worrying” (with a change in BIC of –221.99). ***Table 1*** depicts the change in the BIC value for each arrow.

**Table 1:** Arrows Weight Values in the Directed Acyclic Graphs.


Arrow		Value determining arrow thickness	
	
From	To	BIC	Directional Probability

Nervous	Stop_Worry	–171.33	.51

Nervous	Tr_Relaxing	–25.53	.51

Nervous	Resltess	–6.05	.87

Nervous	Afraid	–21.56	.87

Stop_Worry	Tr_Relaxing	–117.03	.51

Stop_Worry	Restless	–36.60	.82

Stop_Worry	Irritability	–47.08	.52

Stop_Worry	Afraid	–30.93	.66

Worrying	Nervous	–603.89	.51

Worrying	Stop_Worry	–221.99	.51

Worrying	Tr_Relaxing	–286.63	.51

Worrying	Irritability	–48.14	.52

Worrying	Afraid	–48.52	.67

Tr_Relaxing	Restless	–137.70	.83

Tr_Relaxing	Irritability	–84.63	.51

Tr_Relaxing	Afraid	–5.49	.63

Irritability	Restless	–72.23	.80

Irritability	Afraid	–3.02	.67


***Note***: BIC = change in Bayesian Information Criterion when that arrow is removed from the network. BIC values determine arrow thickness in Figure 3A (reflecting the importance of that arrow to the network structure). For the BIC values, negative values correspond to decreases in the network score that would be caused by the arrow’s removal. In other words, negative scores mean that model fit improves with the presence of that arrow. Directional probability values determine arrow thickness in Figure 3B (reflecting the frequency that arrow was present in that direction in the 10,000 bootstrapped networks). Nervous = Feeling nervous, anxious, or on edge (item 1); Stop_Worry = Not being able to stop or control worrying (item 2); Worrying = Worrying too much about different things (item 3); Tr_Relaxing = Trouble relaxing (item 4); Restless = Being so restless that it is hard to sit still (item 5); Irritability = Becoming easily annoyed or irritable (item 6); Afraid = Feeling afraid as if something awful might happen (item 7).

In ***Figure 3*** (Panel B), the thickness of the arrows represents directional probabilities—that is, the proportion of the averaged 10,000 bootstrapped networks wherein that arrow was pointing in that direction. The thickest arrows connect “Nervousness” and “Being Restless” (with a directional probability of .87; i.e., this edge pointed in that direction in 8,700 of 10,000 bootstrapped networks, and in the other direction in only 1,300 of the bootstrapped networks), “Difficulty to Control Worrying” and “Being Restless” (with a directional probability of .83), and “Trouble Relaxing” and “Being Restless” (with a directional probability of .83). The directional probability for each arrow in ***Figure 3*** can be found in ***Table 1***.

Structurally, because DAGs encode the conditional independence relationships and portray the joint probability distribution of each node, the organization of a node within a DAG can be seen as a product of each node’s conditional distribution knowing its parent nodes in the estimated model (McNally, 2021; McNally et al., 2017). Here, because “Worrying Too Much” emerged as the parent node in the model, “Nervousness”, “Trouble Relaxing”, “Difficulty to Control Worrying”, “Irritability”, and “Feeling Afraid that Something Awful Might Happen” are likely to be probabilistically dependent on this parent node, thus suggesting that participants are more prone to exhibit those anxious symptoms if, and only if, they report excessive worrying than the other way around. Likewise, “Trouble Relaxing”, “Being Restless”, and “Feeling Afraid that Something Awful Might Happen” were also probabilistically dependent on “Difficulty to Control Worrying”. Finally, note that “Feeling Afraid that Something Awful Might Happen” and “Being Restless” appeared at the bottom of the probabilistic cascade, thus suggesting, from this computational Bayesian perspective, that they can both be thought of as probabilistically resulting from the other nodes in the model.

## Discussion

Since the WHO declared the COVID-19 pandemic on March 11, 2020, the novel coronavirus, SARS-CoV-2, has profoundly impacted public health and the economy worldwide. But there are not the only ones to be hit. The pandemic also has yielded substantial impacts on mental health, as indexed by global increases in the prevalence and severity of depression, anxiety, and stress symptoms during the pandemic (e.g., Asmundson & Taylor, 2020; Gonzalez-Sanguino et al., 2020; Xiong et al., 2020). The immediate and long-term health, economic, and social consequences of the COVID-19 lockdown and other social distancing measures have been distressing and sources of worries for many people (e.g., Asmundson & Taylor, 2020; Blix et al., 2020; Mertens et al., 2020; Quiu et al., 2020), regardless of whether they have had direct exposure to the virus or not. In this study, our main goal was to clarify how excessive worrying may interact with other hallmark features of anxiety and examine whether it appears especially central to the network organization of anxiety features during the early phase of the first Belgian COVID-19 lockdown-phase. To do so, we computed network analyses by implementing two distinct computational network approaches: a GGM and a DAG.

The outputs of the two computational approaches were remarkably convergent, despite their varying assumptions and constraints. Both the GGM and the DAG pointed to excessive worrying and trouble relaxing as two nodes playing especially influential roles in the network system. First, the two nodes appeared as highly interconnected in the GGM and incident to the thickest and most vital edges in the network (e.g., the edges connecting excessive worrying and trouble relaxing; excessive worrying and nervousness, excessive worrying and uncontrollability of worry, difficulty relaxing and restlessness; trouble relaxing and irritability). Second, when considering the centrality estimates, which provide a fine-grained analysis about which nodes are essential to maintaining the network’s coherence as a whole, excessive worrying and trouble relaxing emerged as the two nodes yielding the highest centrality. Of critical importance, there were significantly more central than all other nodes. Finally, the DAG elucidated the especially intriguing interrelationship between those two nodes. Excessive worrying topped the cascading network of probabilistic dependencies between nodes, whereas trouble relaxing emerged as probabilistically dependent on it. Because excessive worrying appeared at the top of the model, it suggests that, from a probabilistic perspective, people were unlikely to manifest the distinct features of anxiety unless they exhibited excessive worrying.

The most striking point of our findings was their strict alignment with prior research on the network structure of the GAD-7 during the COVID-19 lockdown (i.e., Hoffart et al., 2021; Wang et al., 2020). Such an observation is particularly striking, provided the varying countries, cultural backgrounds, and political responses vis-à-vis the pandemic between these studies and ours. Indeed, Hoffart et al. (2021) reported excessive worry and trouble relaxing as the most central nodes among Norwegian adults during the lockdown’s early weeks. Likewise, Wang et al. (2020) identified excessive worrying, trouble relaxing as the most central nodes in the structure of GAD symptoms among Chinese adults during the first weeks of the lockdown-phase.

Note that Wang et al. (2020) also pointed to the uncontrollability of worry and restlessness as highly central nodes in the network structure in their Chinese sample, and that Hoffart et al. (2021) likewise identified uncontrollability of worry (but not restlessness) as the central nodes in their Norwegian sample. Although uncontrollability of worry was not as central as excessive worrying and trouble relaxing in our study, those two nodes were also highly connected (i.e., high centrality estimates in the GGM and high probabilistic importance in the DAG) in our sample, thus aligning with Hoffart et al. (2021) and Wang et al. (2020). However, in contrast to the study of Wang et al. (2020) among Chinese adults, feeling restlessness did not emerge as a central node in ours. On the other hand, this observation aligns with those of Hoffart et al. (2021) among Norwegian adults tested during the lockdown’s early weeks. One explanation for this discrepancy could be that China had been, during the early phase of the outbreak, much more reactive than the European countries to contain the spread of the pandemic, notably via the implementation of bolder and more stringent (and likely more exhausting) measures than most European countries (e.g., Gibney, 2020; Gu et al., 2020). Future iterations may thus want to clarify whether the centrality of feeling restlessness varies as a function of stringency of the nation-wise implemented measures.

From a theoretical perspective, the observation of a highly central role of excessive worrying in the present study is not surprising. Indeed, for decades, research on anxiety has been emphasizing the determining role of worries in triggering anxiety feelings when one’s concerns are not only broadly diffuse but also future-oriented and related to threats that are not immediately present and may or may not occur—as in the present pandemic context (e.g., worry about the uncertain post-pandemic economic recession, worry about possible job loss, worry about uncertain risks of future COVID-19 resurgence; Blix et al., 2020; Cook & Grimshaw, 2021; Fana, Torrejón Pérez, & Fernández-Macías, 2020; Tamesberger & Bacher, 2020). Moreover, it is worth reminding the assumed adaptive nature of anxiety as an emotion. Indeed, in contrast to fear, anxiety is not a short-lived response (Grillon, 2008; Öhman, 2008). Instead, it is a future-oriented emotion characterized, at the cognitive level, by anticipations of a possible danger that is not present and may never occur (e.g., worry about a potential and uncertain threat, e.g., Grillon, 2008; Öhman, 2008) and, at the physiological level, by physical tension and chronic over-arousal (e.g., trouble relaxing, nervousness, restlessness) thought as reflecting readiness for dealing with a future danger should it occur (APA, 2015; Grillon, 2008; Öhman, 2008).

Although one might view, from a pure emotion neuroscience perspective (e.g., APA, 2015; Grillon, 2008; Öhman, 2008), future-oriented worries as an especially adaptive response given the climate of uncertainty related to the COVID-19 pandemic as it would allow people to plan and prepare for possible but not imminent threat or negative consequences of the pandemic, anxious worries have been identified, in previous epidemics, as highly predictive of long-term maladaptive outcomes, including mental health issues (e.g., Mihashi et al., 2009; Taylor, Agho, Steven, & Raphael, 2008). For instance, worries about future social and economic consequences have been tagged as a risk factor for psychological disorders after (but not during) the SARS-2003 outbreak (e.g., Mihashi et al., 2009). Early results have accordingly suggested that worrying during the lockdown, even when adjusting for many other psychological variables, was significantly associated with a higher level of psychological distress and a lower level of life satisfaction during the later phase of the COVID-19 pandemic (Blix et al., 2020; El-Gabalawy & Sommer, 2021; Elmer et al., 2020; Kampfen et al., 2020).

The present study may yield clinical implications. Network models of psychopathology posit that highly central nodes (i.e., symptoms) are critical to the course of the disorder since, according to the network perspective, if a central symptom is activated, it is more likely to trigger and influence other symptoms (Borsboom, 2017; McNally, 2016; for a discussion, see Blanchard & Heeren, 2022). And, although the very causal involvement of central nodes in determining the network topology remains to be experimentally proven (for discussion, see Blanchard & Heeren, 2022; Bringmann et al., 2019), early results have confirmed the highly predictive nature of highly central nodes in determining the onset, course, and recovery of psychological disorders (e.g., Boschloo et al., 2016; Elliott, Jones, & Schmidt, 2020; Papini, Rubin, Telch, Smits, & Hien, 2020; Spiller et al., 2020). If this holds true for the models investigated in this study, an intervention that would specifically “turn off” excessive worry should then lead to downstream improvement by rendering the entire network less active. In contrast, targeting a peripheral node (e.g., irritability) should have, from this perspective, a much lesser impact. Moreover, because the DAG structure suggests that symptoms of anxiety are less likely in the absence of excessive worries, it also points to this latter as a potential target ripe for prophylactic and therapeutic interventions. Note that, independently from the present study, current clinical research efforts for developing an evidence-based approach for prevention and treatment of the pandemic’s mental consequences have likewise pointed to worries as the top-priority clinical target (e.g., Wahlund et al., 2021). Practitioners may also want to capitalize on previous research efforts to develop clinical interventions for worries and related phenomena (e.g., Heeren & Philippot, 2011; Hoebeke et al., 2021; Newman et al., 2011).

Our results may also yield other implications. Because worrying is assumed to be triggered when facing an unpredictable possible future-oriented threat, the communication strategy of the public health officials and government representatives about not only the pandemic per se but also about the national plan to alleviate the social and financial post-pandemic challenges might also foster excessive worrying and, in turn, anxiety feelings (for a discussion, see Brooks et al., 2020; Frewer, 2003; Xiang et al., 2020). Research indicates that the communication approaches implemented by governments and experts during health and economic crises do influence the perception of (un)predictability and (un)controllability of the situation (e.g., Frewer, 2003; Xiang et al., 2020). Because these latter are known as core driving features of anxious worries and other anxiety-related phenomena, careful communication strategies should thus be implemented by experts and national representatives. Taking stock on previous crises (e.g., Frewer, 2003), transparent communication efforts about the state of knowledge, decision processes, and plan of actions regarding the post-pandemic strategy plan may help to better prepare for the post-crisis world and, in turn, benefit the emotion regulation of future-oriented feelings, like anxiety (for a discussion, see Mheidly & Fares, 2020). Note that the World Health Organization has provided useful tools and guidelines for mass communication in the context of very anxiogenic previous major public health crises (e.g., *http://www.euro.who.int/__data/assets/pdf_file/0004/329647/Vaccines-and-trust.PDF?ua=1*). The OECD has likewise provided relevant recommendations for transparent mass communication in other problematic contexts (*https://www.oecd.org/environment/cc/Enhancing-transparency-climate-change-mitigation-V2.pdf*).

However, these promising results notwithstanding, our approach has limitations. First, one of our study’s main limitations is that the edges’ estimation relies on cross-sectional data, thus excluding any strong inference regarding the potential cause-effect relationships between the seven nodes of the GGM (for discussion, see Blanchard & Heeren, 2022; Maurage, Heeren, & Pesenti, 2013). The only insight into the possible direction of associations is from the DAG, which relies on probabilistic Bayesian learning methods to provide clues about the direction of the associations between the variables (e.g., Heeren et al., 2020; McNally et al., 2017; Moffa et al., 2017). Indeed, a DAG can be seen as the product of each node’s conditional distribution in the model given its parent nodes, thus rendering the DAG capable of indicating whether the presence of node A probabilistically implies node B more than vice versa. However, this direction does not signify the temporal precedence of node B (Bonchi et al., 2017; Pearl, 2009) nor be interpreted as a causal effect (e.g., Heeren et al., 2020; Moffa et al., 2017). Instead, the DAG provides clues about probabilistic dependence between the variables and should be used in a hypothesis-generating fashion rather than a hypothesis-testing one (for discussion, see Pearl, 2009; Rohrer, 2018).

Second, by definition, DAGs assume that connections between nodes are directed and acyclic. Yet, relationships between variables cannot always be defined as directed and acyclic relations of probabilistic dependencies (e.g., in the case of feedback loops). On the other hand, because the direction of the arrow is determined by the percentage of bootstrapped networks wherein this arrow pointed in that direction, one can easily estimate the degree of potential reverse directionality in the DAG from the proportion of the bootstrapped networks wherein the arrow pointed in the other direction (McNally et al., 2017). Here, except a few edges, most were thin, suggesting frequent directional reversals and possible cycles (for a discussion, see McNally et al., 2017). For instance, the edge pointed from Worrying to Trouble Relaxing in only 51% of 10,000 bootstrapped networks, thus indicating that it pointed in the other direction in 49% of the 10,000 bootstrapped networks (see ***Table 1***). The direction of prediction between worrying and trouble relaxing may thus go both ways. Amassing longitudinal data would help to clarify the interplay between variables. Graphical vector autoregressive modeling approaches on intensive time-series data (e.g., 5 to 7 repeated assessments per day over the course of weeks; Aalbers et al., 2019; Contreras et al., 2020; for a review, see Blanchard et al., 2021b) may be especially helpful at revealing feedback loops (Epskamp, 2020).

Third, network analysis, like any statistical approach, only considers variables entered into the model. Although parsimonious networks, like the one presented here, are helpful for hypothesis generation, there could be essential variables left out. For instance, health anxiety, media use, social media use, and risks for loved ones have been identified as key predictors of fear of coronavirus (e.g., Mertens et al., 2020). Likewise, sleep problems can impact excessive worrying and other anxiety-related symptoms (e.g., Coussement & Heeren, 2022). All these variables might thus be at play here, as well, and should therefore be kept in mind when considering the implications of our findings.

Moreover, we assessed anxiety symptoms via the GAD-7. Although it is one of the most commonly used self-reported screening tools in epidemiological and clinical research for assessing GAD symptoms (e.g., Schalet et al., 2014), one may wonder whether the same patterns of findings would emerge using other measurement tools. Although this issue is not confined to GAD research (e.g., Desmedt et al., 2021; Fried, 2017). This point is important since the GAD-7 does not completely overlap with all the criteria for the DSM-5 diagnosis of the GAD (APA, 2013). In particular, two DSM-5 criteria are missing in the GAD-7: sleep problems (criterion GAD C6) and being easily fatigued (criterion GAD C2). Yet, those two criteria are the two ones that are not specific to GAD and overlap with other conditions (e.g., depression; Coussement & Heeren, 2022). Although we focused on the GAD-7 to align with prior research on the network structure of anxiety symptoms during the COVID crisis (i.e., Hoffart et al., 2021; Wang et al., 2020), one may want to ensure that the key role of excessive worrying would thus also appear with other measurements tools. Reassuringly, recent research has confirmed this observation and bolsters our confidence that one can generalize from our findings (e.g., Suen et al., 2021; Taylor et al., 2020).

A fourth limitation concerns the relations between node centrality and node variance. Terluin et al. (2016) found that differential variance across symptoms can distort centrality metrics. That is, a symptom whose variance is minimal (restricted range) is likely to have low values of centrality metrics (McNally, 2021). Here, to address this issue, we computed the correlations between the standard deviation and the centrality estimates of the seven nodes to test whether differences in variances may have distorted conclusions about expected influence estimates. The two-tailed Pearson correlation between the standard deviation and expected influence centrality, *r*(5) = .42, *p* = .35, was not significant. Had a significant correlation emerged, this would suggest that a node’s centrality in the network was affected by its variability. Though nonsignificant, the magnitude of the correlation was yet far from zero and thus calls for careful considerations when interpreting node centrality in the present context. However, the importance of excessive worrying in determining the network structure was also evidenced through node predictability in the GGM and the results of the DAGs, suggesting the robustness of this observation in the present data set.

Finally, a major shortcoming of our study is the absence of pre-pandemic data. This issue is especially relevant as previous network research identified excessive worry as the most central node in the network structure of GAD symptoms in clinical and subclinical samples, regardless of the pandemic context (e.g., Beard et al., 2016; Hsu et al., 2020; Osborn et al., 2020). Moreover, aside from the coronavirus crisis, lots of other stressful events also occurred during the same time frame, including fulminating political tensions in Belgium (Apuzzo & Pronczuk, 2020) and a growing awareness of the impending threats of climate change (Heeren et al., 2021), which can both have impacted the present network structure. One might thus wonder about the specificity of the current observation to the current pandemic. On the other hand, a recent study comparing the network structure of common psychiatric symptoms before (i.e., two and ten years earlier) and after the COVID-19 outbreak among 2,011 Brazilians indicated that, although the network remains stable in the two time-points before the COVID-19 outbreak, excessive worrying was the only symptoms that significantly became more connected with other network’s nodes during the COVID-19 (Suen et al., 2022). Future iterations may thus want to examine the potential change in the network structure one or two years ahead, once the COVID-19 restrictions will no longer be relevant, and test whether excessive worrying would become less influential in the network structure.

In conclusion, the present results are certainly not definitive. Instead, it highlights the utility of thinking anxiety features as nodes interacting within a network system in a pandemic context and pointed to the central role of excessive worrying in this web of interacting nodes. Like other network studies, this study fulfills a valuable niche, wherein exploratory data offer hypothesis-generating clues for later hypothesis-testing and more definitive future agendas.

## Additional File

The additional file for this article can be found as follows:

10.5334/pb.1069.s1 Supplementary materials. The Supplementary materials section contains additional description of the variables reported in this study; additional analyses regarding the accuracy of the edge weights; additional analyses regarding the stability of the centrality metrics.

## Footnotes

Although we initially planned to strictly align with Lits et al. (2020)’s final sample of 1,817 participants that exclude participants with missing values on several variables of interest in the initial study (see our preregistration *https://osf.io/p3v4e*), we decided to rely on the entire dataset available for the GAD-7 with completed demographic characteristics—that is, 2,829 participants. Note that we also re-run our R code on the 1,817 reported in the initial study and found almost identical results to the present ones (with identical conclusions). To maximize the robustness and precision of our results, we decided to report the results with the entire sample size.

We also ran all the analyses without applying the nonparanormal transformation and found nearly identical results for the GGM and the DAGs. In this paper, we reported the results based upon the transformed data to align with the strategy planned in our preregistration.

Note that we did not plan this analysis in our preregistration. However, because predictability across nodes also tells us whether a (part of a) network is primarily determined by itself through strong mutual interactions between nodes (high predictability) or whether it is mainly determined by other factors that are not included in the network (low predictability), we decided to report this analysis.

While the graphical LASSO has emerged as the default network estimation method in psychological sciences, it was optimized in fields outside of psychology with very different needs, such as high-dimensional data sets in which the number of nodes vastly exceeds the number of cases (e.g., genes versus subjects in genomics). Williams and his collaborators have shown that, in low-dimensional data sets (i.e., more participants than nodes; like most psychological data sets), regularizing partial correlation networks via the graphical LASSO returns sparse graphs but does so at the expense of possibly omitting genuine edges (Williams & Rast 2020, Williams et al. 2019). We thus also estimated our GGM network via the ggmModSelect algorithm of the qgraph package. This algorithm searches for an optimal unregularized Gaussian graphical model (GGM) by iteratively changing the initially estimated edges until the Bayesian information criterion (BIC) can no longer be improved (Isvoranu & Epskamp, 2021). In the current data set, we found that this approach leads to findings nearly identical to those estimated via the graphical LASSO algorithm (see Figures S2 and S3 in the supplementary materials).
