# Supplementary material for: Excessive Worrying as a Central Feature of Anxiety during the First COVID-19 Lockdown-Phase in Belgium: Insights from a Network Approach
Source: Psychol Belg. 2021 Dec 30;61(1):401–18. doi: 10.5334/pb.1069 (PMC8719470; doi:10.5334/pb.1069)
Supplement: Supplementary materials. — The Supplementary materials section contains additional description of the variables reported in this study; additional analyses regarding the accuracy of the edge weights; additional analyses regarding the stability of the centrality metrics. [file pb-61-1-1069-s1.pdf]

Supplementary materials

Excessive Worrying as a Central Feature of Anxiety During the First COVID-19 Lockdown-Phase in Belgium: Insights from a Network Approach

Alexandre Heeren[1,2], Bernard Hanseeuw[2,3,4], Louise-Amélie Cougnon[5,6], & Grégoire Lits[6]

[1]Psychological Sciences Research Institute, Université catholique de Louvain, Louvain-la-Neuve, Belgium

[2] Institute of Neuroscience, Université catholique de Louvain, Brussels, Belgium

[3] Neurology Department, Cliniques Universitaires Saint-Luc, Brussels, Belgium

[4] Gordon Center for Medical Imaging, Radiology Department, Massachusetts General Hospital, Harvard Medical School, Boston, MA, USA.

[5] Media Innovation & Intelligibility Lab, Université catholique de Louvain, Louvain-la-Neuve, Belgium

[6] Language and Communication Institute, Université catholique de Louvain, Louvain-la-Neuve, Belgium

Correspondence: alexandre.heeren@uclouvain.be

Additional Descriptions of the Variables

Mean, standard deviation, skewness, and kurtosis are presented separately for each node in *Table S1*. The Pearson product-moment correlations between each pair of variables are plotted in *Figure S1*.

Accuracy of the Edge Weights

With the R package *bootnet* (Epskamp, Borsboom, & Fried, 2018), we bootstrapped confidence regions of the edge weights by using a non-parametric approach and sampling data with 1,000 replacements to estimate the accuracy of the graphical LASSO network. The edges were reasonably stable, and more than 90% of the edges exhibited values significantly different than zero (see *Figure S2)*.

Stability of the Centrality and Bridge Centrality Metrics

We then evaluated the stability of the centrality metrics by implementing a subset bootstrap procedure (Costenbader & Valente, 2003). To do so, we repeatedly correlated the centrality metrics of the original dataset with the metrics calculated from a subsample of participants missing via person-dropping bootstraps as implemented in the R package *bootnet* (Epskamp et al., 2018). If correlation values decline substantially as participants are removed, then this centrality index would be considered less stable. We set the bootstraps to 1,000. Results indicated that both expected influence and bridge expected influences estimated are highly stable (see *Figure S3).* We also calculated the centrality stability correlation coefficient (CS-coefficient) to quantify the effects of this person-dropping procedure. The CS-coefficient represents the maximum proportion of participants that can be dropped while maintaining 95% probability that the correlation between centrality metrics from the full data set and the subset data are at least .70. Based on a simulation study (Epskamp et al., 2018), a minimum CS-

coefficient of .25 (and preferably ≥ .50) is recommended for interpreting centrality indices. In the present dataset, the CS-coefficients were .75 for the expected influence.

References

Costenbader, E., & Valente, T. W. (2003). The stability of centrality measures when networks are sampled. *Social Networks, 25*, 283–307.

Epskamp, S., Borsboom, D., & Fried, E. I. (2018). Estimating psychological networks and their accuracy: A tutorial paper. *Behavior Research Methods, 50*, 195–212.

Table S1. Mean (M), standard deviation (SD), minimum (Min), maximum (Max), skewness, and kurtosis of each node.

**Item**	**M**	**SD**	**Min**	**Max**	**Skewness**	**Kurtosis**
Feeling nervous, anxious, or on edge (item 1)	.65	.92	0	3	1.34	.81
Not being able to stop or control worrying (item 2)	.48	.77	0	3	1.71	2.52
Worrying too much about different things (item 3)	1.08	.98	0	3	.72	-.42
Trouble relaxing (item 4)	.80	.98	0	3	.72	.42
Being so restless that it is hard to sit still (item 5)	.50	.79	0	3	1.70	2.36
Becoming easily annoyed or irritable (item 6)	.72	.84	0	3	1.15	.82
Feeling afraid as if something awful might happen (item 7)	.69	.84	0	3	1.23	.97

Figure S1. Pearson product-moment correlations between each GAD symptoms.

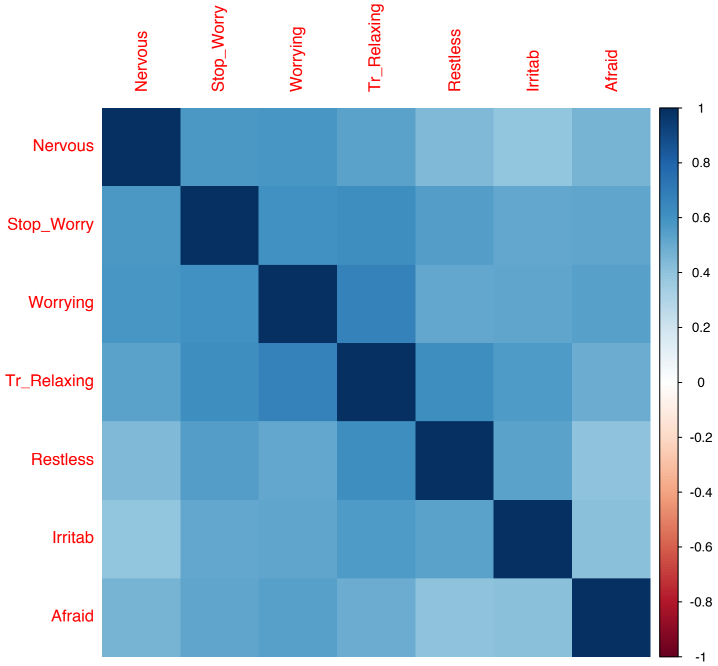

Note. Nervous = Feeling nervous, anxious, or on edge (item 1); Stop_Worry = Not being able to stop or control worrying (item 2); Worrying = Worrying too much about different things (item 3); Tr_Relaxing = Trouble relaxing (item 4); Restless = Being so restless that it is hard to sit still (item 5); Irritab = Becoming easily annoyed or irritable (item 6); Afraid = Feeling afraid as if something awful might happen (item 7).

Figure S2. Graphical Gaussian Model Constructed via the ggmModSelect algorithm that searches for an optimal unregularized Gaussian graphical model by iteratively changing the initially estimated edges until the Bayesian information criterion.

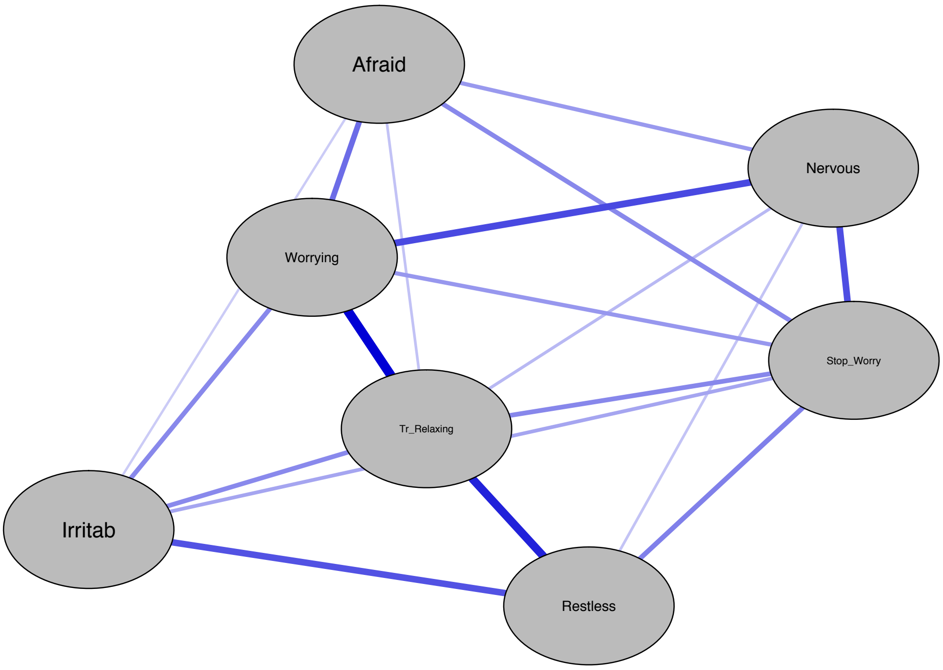

Note. The thickness of an edge reflects the magnitude of the association (the thickest edge representing a value of .33). Nervous = Feeling nervous, anxious, or on edge (item 1); Stop_Worry = Not being able to stop or control worrying (item 2); Worrying = Worrying too much about different things (item 3); Tr_Relaxing = Trouble relaxing (item 4); Restless = Being so restless that it is hard to sit still (item 5); Irritab = Becoming easily annoyed or irritable (item 6); Afraid = Feeling afraid as if something awful might happen (item 7).

Figure S3. Expected Influence Estimates of the Graphical Gaussian Model Constructed via the ggmModSelect algorithm

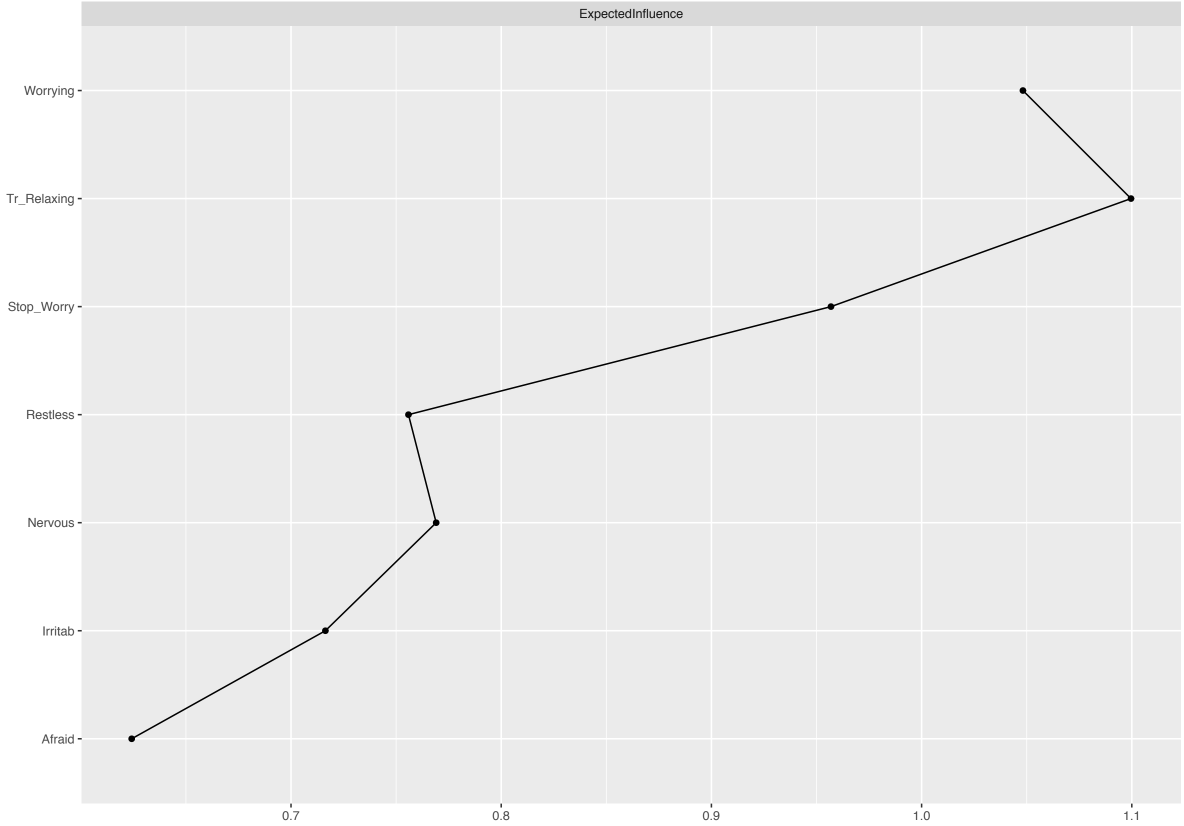

Note. Nervous = Feeling nervous, anxious, or on edge (item 1); Stop_Worry = Not being able to stop or control worrying (item 2); Worrying = Worrying too much about different things (item 3); Tr_Relaxing = Trouble relaxing (item 4); Restless = Being so restless that it is hard to sit still (item 5); Irritab = Becoming easily annoyed or irritable (item 6); Afraid = Feeling afraid as if something awful might happen (item 7).

Figure S4. Bootstrapped confidence intervals of estimated edge weights for the graphical lasso network.

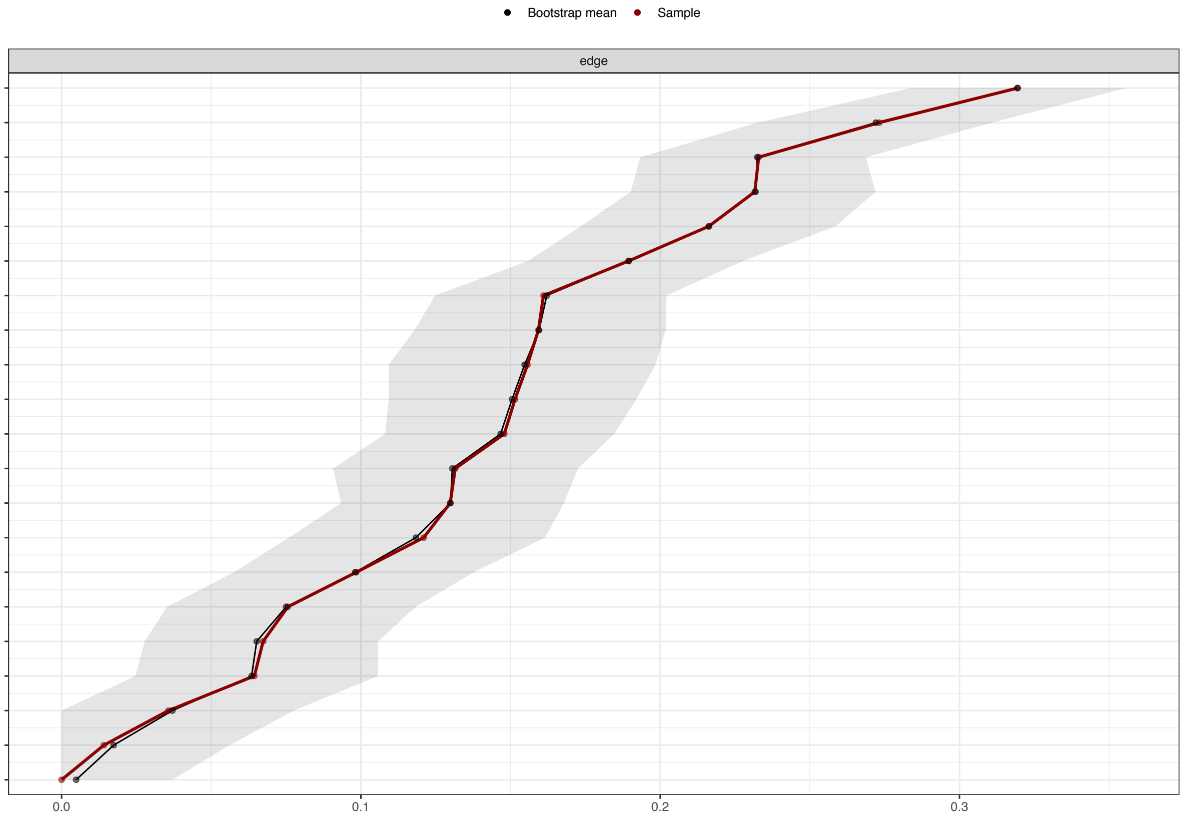

Note. The red line indicates the sample values and the gray area the 95% confidence intervals. The dark line indicates the bootstrapped mean values.

Figure S5. Bootstrapped difference tests (α = 0.05) between edge-weights that were non-zero in the graphical LASSO network.

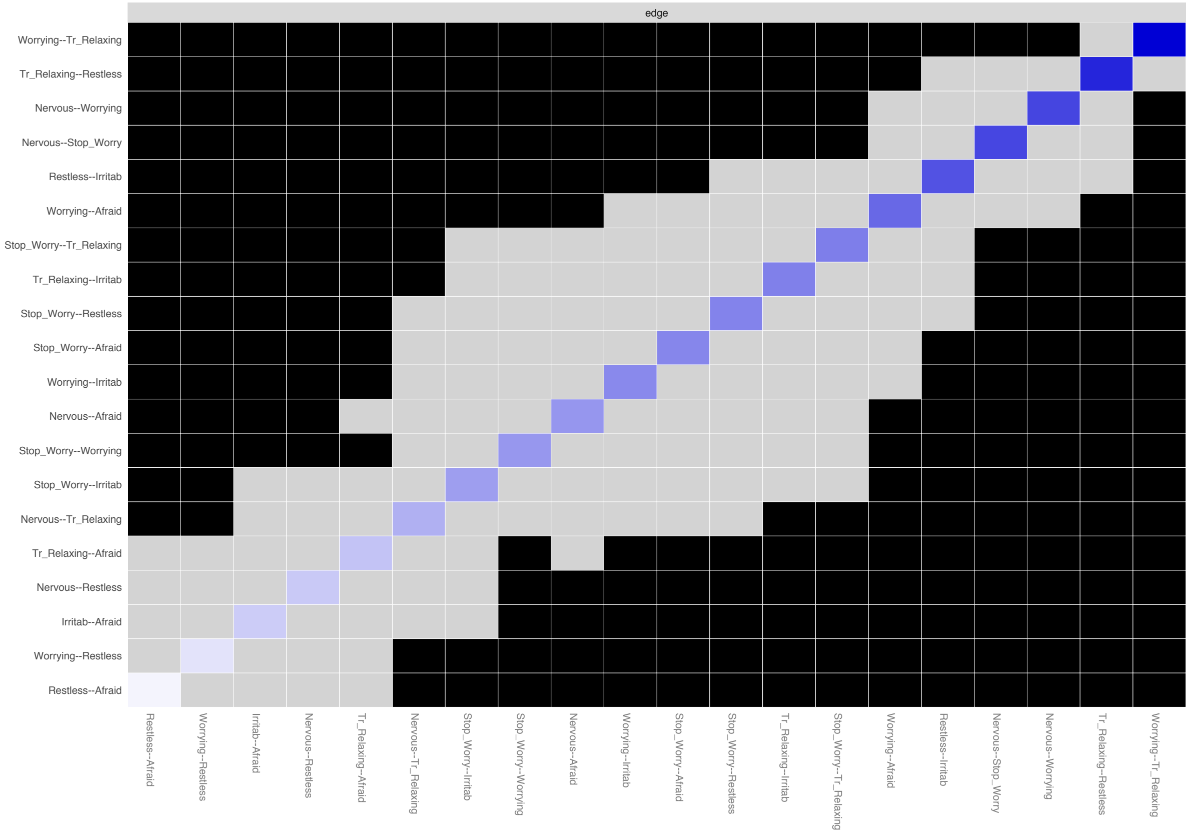

Note. Gray boxes indicate edges that do not differ significantly from one another and black boxes represent edges that do differ significantly from one another. Colored boxes correspond to the magnitude of the difference, with bluer color denoting larger difference. Nervous = Feeling nervous, anxious, or on edge (item 1); Stop_Worry = Not being able to stop or control worrying (item 2); Worrying = Worrying too much about different things (item 3); Tr_Relaxing = Trouble relaxing (item 4); Restless = Being so restless that it is hard to sit still (item 5); Irritab = Becoming easily annoyed or irritable (item 6); Afraid = Feeling afraid as if something awful might happen (item 7).

Figure S6. Average correlation between centrality indices (i.e., expected influence) of the network estimation sampled with persons dropped and the original sample.

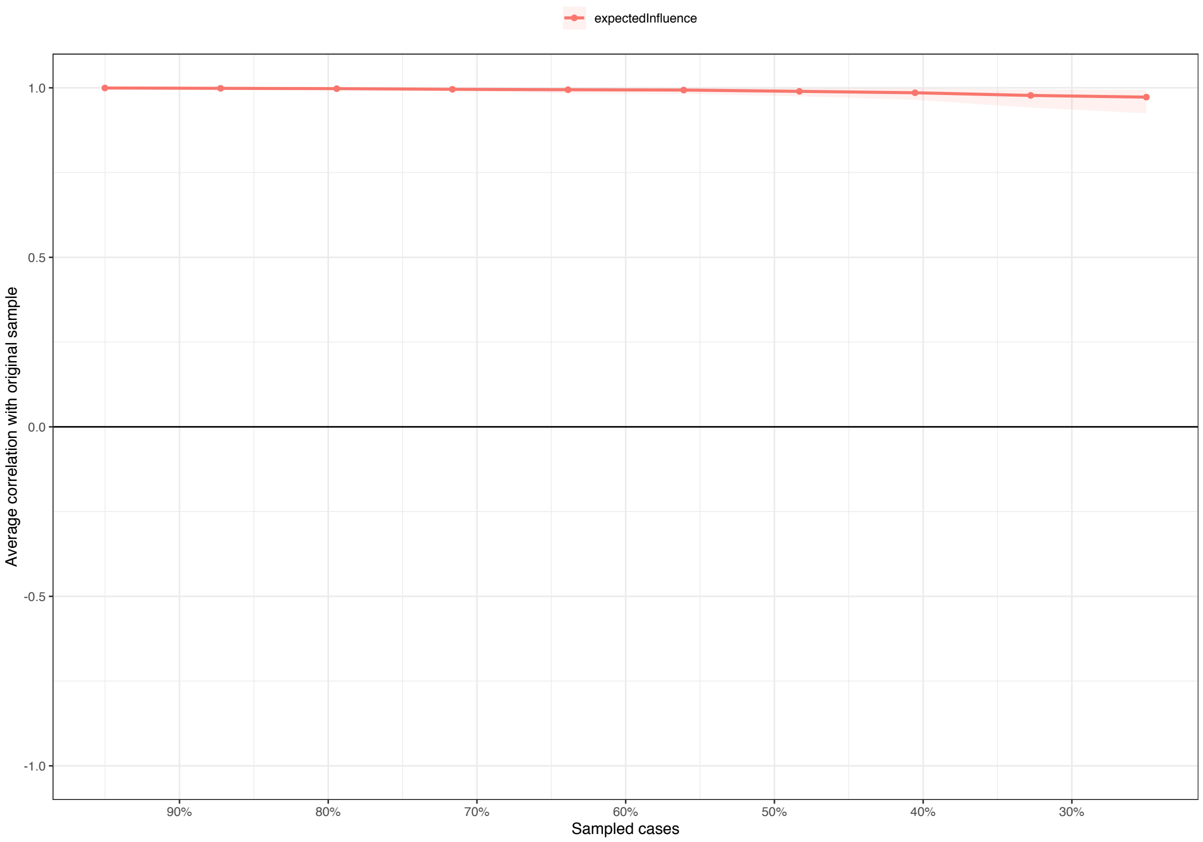

Figure S7. *Bootstrapped difference test between node expected influence*

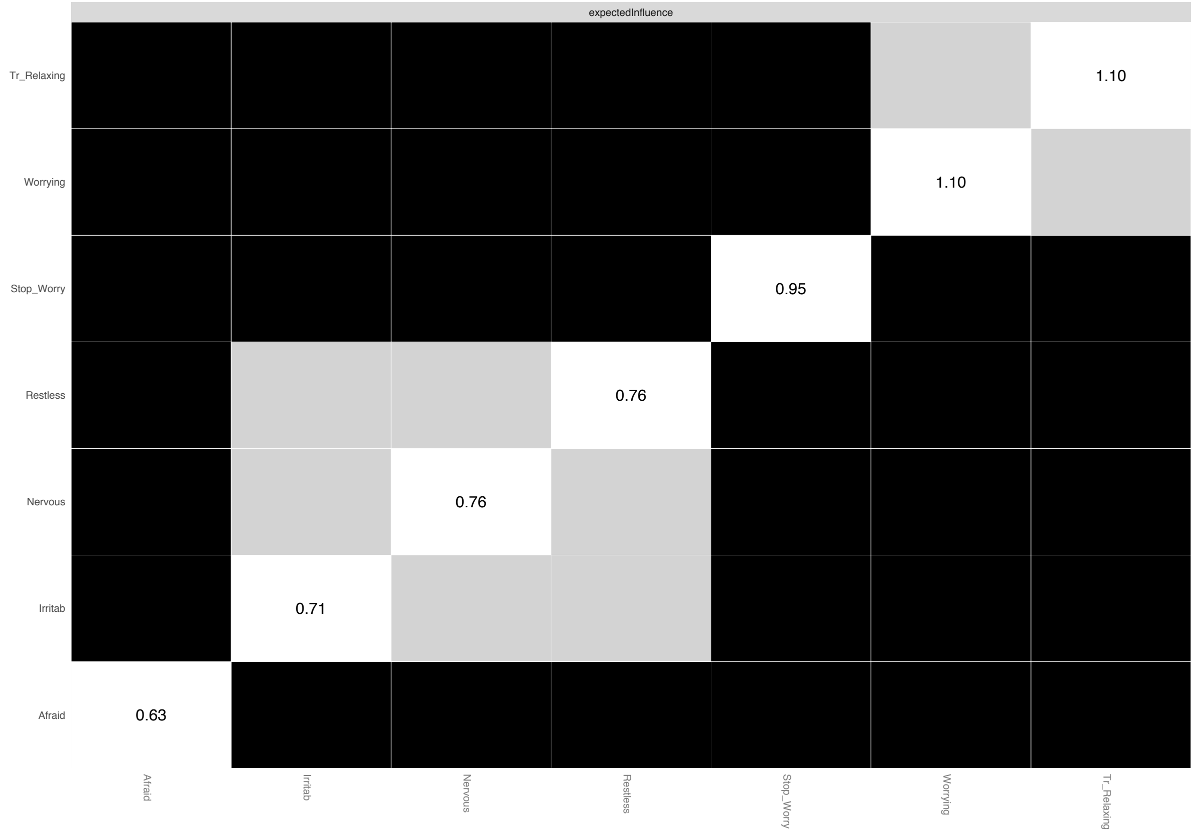

Note. Black boxes represent nodes that do differ significantly from one another and gray boxes indicate nodes that do not differ significantly from one another. Nervous = Feeling nervous, anxious, or on edge (item 1); Stop_Worry = Not being able to stop or control worrying (item 2); Worrying = Worrying too much about different things (item 3); Tr_Relaxing = Trouble relaxing (item 4); Restless = Being so restless that it is hard to sit still (item 5); Irritab = Becoming easily annoyed or irritable (item 6); Afraid = Feeling afraid as if something awful might happen (item 7).
